# Different contribution of extent of myocardial injury to left ventricular systolic and diastolic function in early reperfused acute myocardial infarction

**DOI:** 10.1186/1476-7120-12-6

**Published:** 2014-02-10

**Authors:** Hyemoon Chung, Ji-Hyun Yoon, Young Won Yoon, Chul Hwan Park, Eun Jung Ko, Jong Youn Kim, Pil-Ki Min, Tae Hoon Kim, Byoung Kwon Lee, Bum-Kee Hong, Se-Joong Rim, Hyuck Moon Kwon, Eui-Young Choi

**Affiliations:** 1 Division of Cardiology, Heart Center, Gangnam Severance Hospital, Yonsei University College of Medicine, Seoul, Republic of Korea; 2 Department of Radiology, Gangnam Severance Hospital, Yonsei University College of Medicine, Seoul, Republic of Korea

**Keywords:** Acute myocardial infarction, Diastolic function, Cardiac magnetic resonance, Speckle tracking echocardiography

## Abstract

**Background:**

We sought to investigate the influence of the extent of myocardial injury on left ventricular (LV) systolic and diastolic function in patients after reperfused acute myocardial infarction (AMI).

**Methods:**

Thirty-eight reperfused AMI patients underwent cardiac magnetic resonance (CMR) imaging after percutaneous coronary revascularization. The extent of myocardial edema and scarring were assessed by T2 weighted imaging and late gadolinium enhancement (LGE) imaging, respectively. Within a day of CMR, echocardiography was done. Using 2D speckle tracking analysis, LV longitudinal, circumferential strain, and twist were measured.

**Results:**

Extent of LGE were significantly correlated with LV systolic functional indices such as ejection fraction (r = -0.57, p < 0.001), regional wall motion score index (r = 0.52, p = 0.001), and global longitudinal strain (r = 0.56, p < 0.001). The diastolic functional indices significantly correlated with age (r = -0.64, p < 0.001), LV twist (r = -0.39, p = 0.02), average non-infarcted myocardial circumferential strain (r = -0.52, p = 0.001), and LV end-diastolic wall stress index (r = -0.47, p = 0.003 with e’) but not or weakly with extent of LGE. In multivariate analysis, age and non-infarcted myocardial circumferential strain independently correlated with diastolic functional indices rather than extent of injury.

**Conclusions:**

In patients with timely reperfused AMI, not only extent of myocardial injury but also age and non-infarcted myocardial function were more significantly related to LV chamber diastolic function.

## Introduction

The hemodynamics on infarcted or non-infarcted myocardium is related to left ventricular (LV) remodeling after acute myocardial infarction (AMI) [1]. Post-myocardial infarction remodeling develops both in the infarcted and remote myocardium, so called “infarct expansion and LV dilatation” [2,3]. This remodeling process can induce heart failure through systolic dysfunction or advanced diastolic dysfunction. However, the influence of myocardial tissue characteristics after reperfused AMI on regional or global myocardial function has not been fully investigated. Classically, the extent of myocardial infarction has been accepted as a main determinant of LV systolic function and future ventricular remodeling [3]. Moreover, both myocardial fibrosis and edema, which may develop as a result of infarct-related damage, have been shown to slow myocardial relaxation and increase myocardial stiffness [4,5]. However, it is not known whether the extent of injury (scarring and edema) mainly determines LV diastolic function. This is especially important in the era of early revascularization such as primary percutaneous coronary intervention. We raised a question whether there are any other important contributing factors than extent of myocardial infarction such as non-infarcted myocardial characteristics or function, which may play a role. These concerns are important to address because LV diastolic dysfunction is a strong prognostic factor after AMI, especially in cases of preserved LV ejection fraction [6]. Therefore, this study uses cardiac magnetic resonance imaging (CMR) to investigate the extent of myocardial injury and speckle tracking echocardiography to measure LV chamber and regional myocardial function.

## Methods

### Study subjects

Patients with AMI who underwent successful percutaneous coronary intervention (PCI) within 48 hours of chest pain were prospectively enrolled. AMI was diagnosed on the basis of elevated levels of cardiac enzyme and ST-segment or T wave deviation on electrocardiography (ECG) according to the established diagnostic criteria [7]. Exclusion criteria were as follows: patients with a previous history of myocardial infarction, claustrophobia, estimated glomerular filtration rate < 30 ml/min, valvular heart disease more than a moderate degree, underlying cardiomyopathies, a cardiac implantable device except for coronary stents, or with poor quality of late gadolinium enhancement (LGE) or T2 weighted images (T2WI). Consecutive patients were enrolled, and four patients were excluded due to claustrophobia (n = 1), denial of enrollment of study (n = 2) or poor breath hold (n = 1). Finally, a total of 38 subjects were studied. CMR was done on average of 2.4 ± 2.7 days after admission and all patients underwent study echocardiography within a day of CMR. Daily electrocardiography follow-up was conducted and cardiac biomarkers were assessed after admission. The extent of Q-wave was calculated by summing all Q-wave depths (mm) from the 12 leads. The study protocol was approved by the institutional review board of Gangnam Severance Hospital (3-2011-0203) and informed consent was obtained by the participants.

#### Cardiac magnetic resonance

Cardiac MRI was performed with a 1.5-T scanner (Magnetom Avanto®; Siemens Medical Solutions, Erlangen, Germany) with a phased array body coil. The LV 2-chamber, 4-chamber, and short axis views were obtained using cine images with steady-state free precession sequence. The acquisition parameters were: repetition time (TR) = 55 msec, echo time (TE) = 1.1 msec, flip angle = 67°, 25 phases, slice thickness = 8 mm, slice gap = 2 mm, acquisition matrix = 192 × 109, and field of view = 320 × 400 mm. T2WI was performed in cardiac short-axis direction using a dark-blood T2-weighted short-tau inversion-recovery fast-spin echo sequence. Imaging parameters were TR of two heart beats; inversion time = 170 msec; TE = 47 msec; flip angle = 180°; turbo factor = 33; matrix = 119 × 256; field of view = 340 × 400 mm; slice thickness = 8 mm. LGE imaging with a magnitude- and phase-sensitive inversion recovery prepared fast gradient echo sequence was obtained in 10 minutes after administration of 0.2 mmol/kg of a gadolinium-based contrast agent (gadoterate dimeglumine; Dotarem, Guerbet, France). LGE imaging was obtained in the same axis and slice thickness used in the cine imaging. A bolus of contrast media was intravenously administered at 2 mL/sec, followed by 20 mL of normal saline at 4 mL/sec through a 20-gauge cannula in the antecubital vein using a power injector (Nemoto; Nemoto Kyorindo, Tokyo, Japan). The appropriate inversion time before LGE-imaing was determined using a fast gradient echo sequence with inversion times varying from 150-650 msec to null the signal from the normal myocardium. The LGE imaging parameters were: TR = 600 msec, TE = 3.4 msec, flip angle = 25°, acquisition matrix = 256 × 156; and field of view = 320 × 400 mm.

### LV geometry and chamber performance assessment

The endocardial and epicardial borders were contoured using a semi-automated method (Argus®, Siemens, Germany), then LV end-diastolic volume (LVEDV) and LV end-systolic volume were measured. To determine the end-diastolic LV mass the difference between the epicardial and endocardial areas for all slices was multiplied by the slice thickness and section gap and then multiplied by the specific gravity of the myocardium (1.05 g/mL). Papillary muscle mass was included in the LV cavity and excluded from the LV mass measurements. Stroke volume was calculated as LV end-diastolic volume (LVEDV) minus end-systolic volume, and LV ejection fraction was calculated as (100 × stroke volume)/LVEDV. LV mass index was calculated by LV mass/body surface area. The LV-end diastolic wall stress (LVEDWS, unit KPa) was calculated as (estimated LV end-diastolic pressure)/{[(LVEDV + LV mass volume)/LVEDV]^2/3^ – 1} from LaPlace’s law [8], where estimated LV end-diastolic pressure was calculated from the equation of [1.9 + 1.24 × (E/e’)] [9]. Where E means early trans-mitral inflow velocity (cm/s) from pulsed wave Doppler images and e’ means early septal mitral annular velocity (cm/s) from tissue Doppler images.

### Extent of LGE and edema

**Figure 1:**
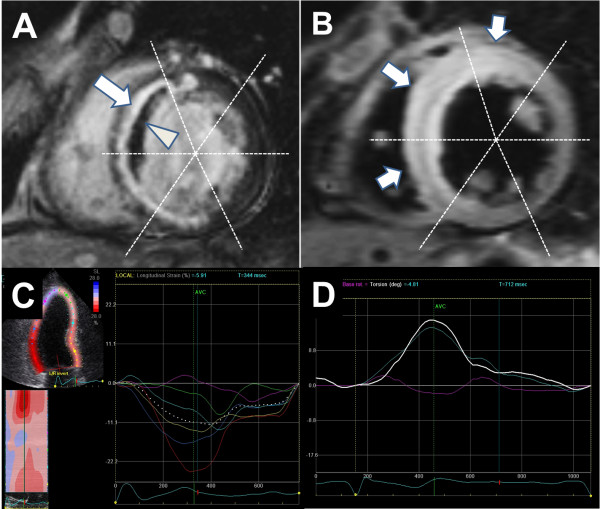
**Measurement of extent of myocardial injury and myocardial function.** Segmentation of left ventricle when measuring extent of myocardial injury from late gadolinium enhancement imaging **(A)** and T2 weighted imaging **(B)**. Representative images of measuring global longitudinal strain **(C)** and twist **(D)** from speckle tracking echocardiography. Arrow in **(A)** indicates LGE and arrow head indicates microvascular obstruction. Arrows in **(B)** indicate higher signal intensity which represents myocardial edema.

From the LGE images, LV was divided into 17 segments as recommended by American Heart Association [10]. In each segment the degree of LGE involvement was measured. The transmural extent of involvement of the LGE was semi-quantitatively measured as 0%, 1-25%, 26-50%, 51-75%, and 76-100% [11]. Lesion of microvascular obstruction (MVO) was included as LGE area. These measurements were then scored as 0, 1, 2, 3 and 4, respectively. The extent of myocardial scarring was defined as the summation of LGE scores from all segments. In addition, the absolute amount of LGE and percentage of LGE were measured using dedicated quantitative analysis software (QmassMR, Medis, Leiden, Netherland). In each short-axis slice image, boundaries of contrast-enhanced areas were automatically traced (using a full-width at half maximum method that defines the enhanced area by using 50% of the maximum signal found within the enhanced area). The maximum signal was determined by computer-assisted window thresholding of the enhanced area. Obvious artifacts such as those caused by motion were excluded by highlighting them using a tool from the software package. Other small isolated regions of enhancement that were clearly not of ischemic origin were also excluded from analysis Total infarct size was calculated by summation of all slice volumes of enhancement [12]. Using the T2WI, the number of edema-involved segments was measured. In T2WI, 17-segment based transmural extent of involvement was not measured due to an unclear border delineation of increased signal intensity, and a 16-segment model was used (Figure 1).

### Conventional echocardiography

Each patient underwent a complete standard transthoracic echocardiography. The LV volume and LV ejection fraction were measured by the biplane Simpson’s method as recommended by the American Society of Echocardiography [13]. The left atrial volume was measured using the prolate ellipsoidal method at the point of LV end-systole when the left atrial size was maximum [14]. Regional wall motion score index (RWMSI) were calculated as sum of wall motion scores divided by number of visualized segment (from 17-segment model), where 1 indicates normal; 2, hypokinesis; 3, akinesis; and 4, dyskinesis. (13) From the apical window, a 1 mm pulsed Doppler sample volume was placed at the mitral valve tip and mitral flow velocities from 5 to 10 cardiac cycles were recorded. Then E and late (A) mitral inflow velocity were measured. Mitral annular velocity was measured by tissue Doppler imaging using the pulsed wave Doppler mode. The filter was set to exclude high frequency signal, and the Nyquist limit was adjusted to a range of 15 to 20 cm/s. Gain and sample volume were minimized to allow for a clear tissue signal with minimal background noise. The e’ and late diastolic velocities of the mitral annulus were measured from the apical 4-chamber view with a 2- to 5-mm sample volume placed at the septal corner of the mitral annulus.

### Speckle tracking echocardiography

For the LV speckle tracking analysis, three parasternal short axis images (base, mid, and apical slices), apical four- and two-chamber view images were obtained using conventional gray scale echocardiography (Vivid 7 or E9; GE Medical Systems, Milwaukee, WI). A minimum frame rate of 40 fps was required for the reliable operation of this program. Recordings were processed using acoustic-tracking software (EchoPAC PC, GE Medical Systems, Milwaukee, WI) that allowed offline, semi-automated analysis of speckle-based strain [14]. From the three parasternal short axis views, segmental and global circumferential strain (GCS) and strain rate (GCSr) values were generated. Non-infarcted myocardial CS was calculated as average of GCS of non-LGE slices. Rotation curves of basal and apical slices were generated followed by twist curves. Peak twist value, systolic and diastolic twist rates were then measured. From the apical 4- and 2-chamber views, global longitudinal strain (GLS) and strain rate (GLSr) curves were generated, and the average of peak value from each curve was used [15]. In ten patients, GCS(r) and GLS(r) and twist (rates) measurements were blindly repeated by two investigators to see the reproducibility.

### Extent of myocardial injury, LV systolic and diastolic functional indicies

Total LGE score, percent LGE and number of high signal intensity in T2WI-CMR were used as an index of extent of LV myocardial infarction and extent of myocardial edema, respectively. As representative LV chamber systolic functional indices, LV ejection fraction, RWMSI, GLS, and GCS were used and as representative diastolic functional indices, E/e’, e’, LVEDWS, early diastolic-GLSr and early diastolic-GCSr by speckle tracking echocardiography were used.

### Statistical analysis

Clinical characteristics, echocardiographic, and CMR parameters are presented as mean ± standard deviation for continuous variables and number (percentage) for categorical variables. Correlation analysis was done between continuous variables with Pearson correlation coefficient. Intraclass correlation coefficients from average measures are calculated for repeated measured strain, strain rate and twist values. For the analysis of predictive value of diastolic function included age, gender, presence of diabetes, hypertension, percent LGE, LV ejection fraction, LV mass index, M/V ratio, LVEDWS, Twist, and non-infarcted myocardial CS. Among them dichomatous variables were used in gender, presence of diabetes and hypertension. Variables with a p < 0.05 in the univariable analysis were included in the stepwise forward method in the multivariable regression analysis. All the analyses were done using SPSS (version 18.0, IBM, USA), and P values less than 0.05 were considered as significant.

## Results

### Baseline characteristics

The mean age was 52.8 ± 11.8 years, and 35 (92%) were men. Thirty patients presented with ST-elevation myocardial infarction (STEMI), and eight were non-STEMI. The average time from onset of chest pain to PCI was 207.4 minutes, and the mean duration of performing CMR after PCI was 2.4 days. Twenty patients had left anterior descending artery territory lesions, three had left circumflex coronary artery territory lesions, and 15 had right coronary artery territory lesions. The mean Killip classification on admission was 1.7 ± 0.9 and the mean NYHA functional class at CMR was 1.4 ± 0.6.Baseline clinical characteristics are described in Table 1. Mean LV mass index was 80.3 ± 21.5 g/m^2^ and LV ejection fraction was 53.0 ± 10.8% as assessed by CMR. The number of patients with an LV ejection fraction of less than <50% was 14 (37%), and 3 (8%) patients had an LV ejection fraction of less than 35%. Mean value of peak CK-MB was 122.8 ± 88.8 ug/L. The number of LGE-involved segments was 4.5 ± 2.4 and the average LGE amount was 21.1 ± 13.9 g (15.0 ± 9.0% of total LV mass). Seventeen patients (45%) had MVO. Percent LGE) was higher in the STEMI group (15.4 ± 9.7% vs. 12.9 ± 5.6% p = 0.500), while the time to reperfusion was longer in the non-STEMI group (352.5 ± 301.8 min vs. 163.7 ± 170.3 min, p = 0.127); nevertheless, this was not significant.

**Table 1:** Baseline clinical characteristics

**Variables**	
Age, years	52.8 ± 11.8
Male, n(%)	35 (92)
LAD/LCx/RCA territory, n	20/3/15
Peak CK level, IU	1782.7 ± 1385.7
Peak CK-MB level, ug/L	122.8 ± 88.8
Peak troponin T level, ug/L	3.62 ± 3.23
Hypertension, n(%)	18 (47)
Diabetes, n(%)	8 (21)
Smoking status (Non-/Ex-/ Current), n	14/10/14
Body surface area, m^2^	1.80 ± 0.20
Systolic blood pressure at CMR, mmHg	113.2 ± 14.3
Diastolic blood pressure mmHg at CMR, mmHg	72.4 ± 9.1
Heart rate, bpm	74.6 ± 11.7

LAD = left anterior descending artery; LCx = left circumflex artery; RCA = right coronary artery; CK-MB = creatinine kinase-MB; CMR = cardiac magnetic resonance.

### Echo-Doppler parameters and myocardial deformation indices

The average e’ velocity was 6.7 ± 2.7 cm/s, E/A ratio was 1.03 ± 0.35 and E/e’ was 11.4 ± 4.7. GLS was -13.2 ± 3.9%. Systolic and early diastolic GLSr was -0.73 ± 0.23 ^1^/s and 0.86 ± 0.34 ^1^/s, respectively. The average value of twist was 17.9 ± 8.5°. The mean LV end-diastolic wall stress was 3.8 ± 1.4 kPa. The details of tissue Doppler indices and 2D speckle tracking echo results are described in Table 2. Six (16%) patients had normal filling pattern, 21 (55%) had relaxational abnormality and 11 (29%) had pseudonormal filling pattern. There was a trend of increase in LGE amount accordingly but it was not statistically significant. (11.9 ± 6.1%, 13.0 ± 7.7% and 20.1 ± 10.9%, p = 0.097 by trend).

**Table 2:** Cardiac magnetic resonance imaging and echocardiographic parameters

**Variables**	
LV end-diastolic volume by CMR, mL	138.7 ± 28.1
LV ejection fraction by CMR, %	53.0 ± 10.8
LV mass index by CMR, g/m^2^	80.3 ± 21.5
LV end-diastolic wall stress, kPa	3.83 ± 1.39
Tissue characterization	
Number of LGE segments (among 17 segments)	4.5 ± 2.4
Number of edema segments (among 16 segments)	4.1 ± 2.7
Sum of LGE score	13.0 ± 9.1
Presence of MVO, n (%)	17 (45)
LGE amount (g)	21.1 ± 13.9
Percent LGE (%)	15.0 ± 9.0
E velocity, cm/s	69.6 ± 21.0
E/A ratio	1.03 ± 0.35
e’ velocity, cm/s	6.71 ± 2.70
E/e’	11.4 ± 4.7
Left atrial volume index, ml/m^2^	21.1 ± 7.0
GLS, %	-13.2 ± 3.9
Systolic GLSr, 1/s	-0.73 ± 0.23
Early diastolic GLSr, 1/s	0.86 ± 0.34
Average GCS, %	-15.8 ± 6.3
GCS-basal slice, %	-13.8 ± 5.4
GCS-midventricular slice, %	-13.6 ± 6.3
GCS-apical slice, %	-20.2 ± 11.5
Average systolic GCSr, 1/s	-1.01 ± 0.45
Systolic GCSr-basal slice, 1/s	-0.86 ± 0.35
Systolic GCSr-midventricular slice, 1/s	-0.89 ± 0.39
Systolic GCSr-apical slice, 1/s	-1.31 ± 0.87
Average early diastolic GCSr, 1/s	1.18 ± 0.64
Early diastolic GCSr-basal slice, 1/s	0.88 ± 0.48
Early diastolic GCSr-midventricular slice, 1/s	0.93 ± 0.52
Early diastolic GCSr-apical slice, 1/s	1.79 ± 1.30
Twist, °	17.9 ± 8.5

LV = left ventricular, EF = ejection fraction, MVO = microvascular obstruction, GLS = global longitudinal strain, GLSr = global longitudinal strain rate, GCS = global circumferential strain, GCSr = global circumferential strain rate.

### Extent of myocardial injury and myocardial function

**Figure 2:**
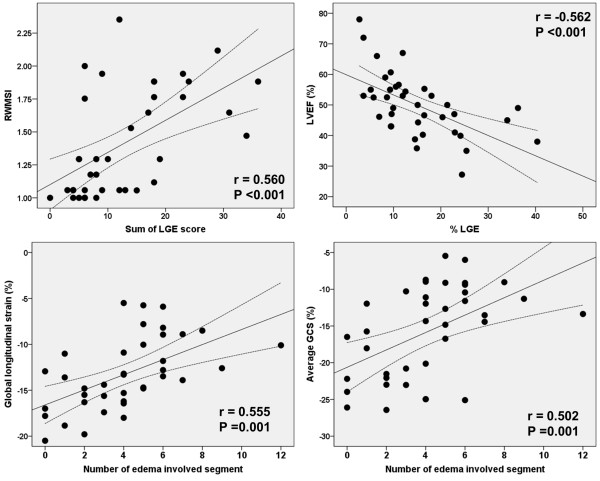
**Relationship between extent of myocardial injury and left ventricular systolic functional indices.** LGE = late gadolinium enhancement; RWMSI = regional wall motion score index.

Total LGE score was significantly correlated with peak CK-MB and peak troponin T, biomarkers of myocardial injury (r = 0.600, p < 0.001 with CK-MB; r = 0.567, p < 0.001 with troponin T) and extent of Q wave. Total LGE score was significantly correlated with LV systolic function as measured by ejection fraction (r = -0.570, p < 0.001) and RWMSI (r = 0.560, p < 0.001). Their relationships remained significant wih%LGE (r = -0.579, p < 0.001 with LVEF; r = 0.562, p < 0.001 with RWMSI). The correlation was also significant with the number of edema-involved segments (r = 0.618, p < 0.001 with RWMSI). The number of edema-involved segments was significantly correlated with GLS (r = 0.555, p < 0.001), the average of three GCSs (r = 0.502, p = 0.001), and the systolic twist rate (r = 0.432, p = 0.025). These relationships were also significant with total LGE score. However the extent of edema did not correlate with peak twist (r = -0.077, p = 0.648) and only weakly correlated with early diastolic twist rate (r = -0.378, p = 0.052). Both the extent of LGE and %LGE were not significantly correlated with diastolic functional parameters. (Figure 2 and Table 3) Diastolic function was not significantly different between patients with MVO and without MVO. (11.4 ± 5.5 vs. 11.3 ± 3.6 p = 0.955 with E/e’; 6.5 ± 2.4 vs. 6.9 ± 3.0 cm/s, p = 0.634 with e’) Age did not correlate with LV systolic functional indices measured by LV ejection fraction (r = -0.075, p = 0.655), RWMSI (r = -0.062, p = 0.713), GLS (r = 0.055, p = 0.743), or average GCS (r = 0.182, p = 0.275) in this study.

**Table 3:** Relationship between extent of myocardial injury and LV systolic or diastolic functional indicies

	**Extent of myocardial injury**			
	**Extent of LGE**	**Extent of edema**	**% LGE**	**Extent of Q-wave**
**Systolic functional index**				
**LV ejection fraction**	-0.57 (<0.001)	-0.54 (<0.001)	-0.58 (<0.001)	-0.39 (0.017)
**RWMSI**	0.52 (0.001)	0.58 (<0.001)	0.57 (<0.001)	0.44 (0.006)
**GLS**	0.42 (0.009)	0.56 (<0.001)	0.36 (0.03)	0.52 (0.001)
**GCS**	0.38 (0.019)	0.50 (<0.001)	0.29 (0.08)	0.36 (0.03)
**Systolic twist rate**	0.22 (0.28)	0.43 (0.03)	0.32 (0.11)	0.43 (0.03)
**Diastolic functional index**				
**E/A ratio**	0.05 (0.78)	-0.02 (0.90)	0.20 (0.23)	0.10 (0.55)
**E’**	-0.27 (0.11)	-0.16 (0.35)	-0.07 (0.70)	-0.08 (0.61)
**E/e’**	0.16 (0.35)	0.11 (0.50)	0.12 (0.49)	-0.12 (0.47)
**LA volume index**	-0.11 (0.52)	-0.20 (0.24)	-0.09 (0.61)	-0.24 (0.15)
**ED-GLSr**	-0.14 (0.40)	-0.17 (0.31)	-0.06 (0.74)	-0.26 (0.12)
**ED-GCSr**	-0.23 (0.17)	-0.24 (0.15)	-0.05 (0.79)	-0.18 (0.27)
**ED-twist rate**	-0.21 (0.31)	-0.38 (0.05)	-0.28 (0.16)	-0.28 (0.17)
**Global functional index**				
**Twist**	-0.21 (0.20)	-0.08 (0.65)	0.03 (0.85)	-0.09 (0.60)

RWMSI = regional wall motion score index, ED = early diastolic, see abbreviations in Table 2.

### Determinants of diastolic function

**Figure 3:**
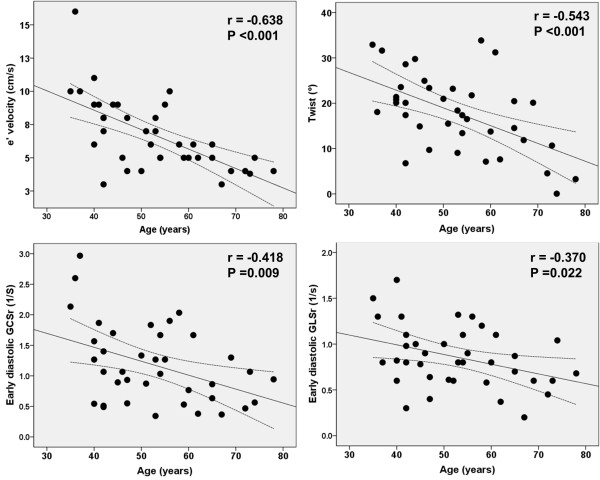
Relationship between age and left ventricular functional indices.

**Figure 4:**
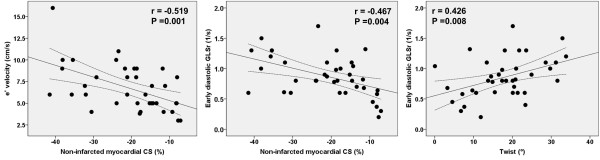
Correlates of left ventricular diastolic functional indices.

In contrast to systolic function, age was significantly correlated with LV chamber diastolic functional indices as measured by e’ (r = -0.638, p < 0.001), E/e’ (r = 0.517, p = 0.001), early diastolic GLSr (r = -0.370, p = 0.022), and average early diastolic GCSr (r = -0.418, p = 0.009). In addition, age was significantly correlated with LV twist (r = -0.543, p < 0.001) and twist significantly correlated with diastolic functional indices such as E/e’, e’, early diastolic GLSr, and average early diastolic GCSr. (Figure 3) The average GCS of the non-infarcted area was significantly correlated with diastolic functional parameters as measured by e’, E/e’, early diastolic GLSr, average early diastolic GCSr, and LVEDWS. (Figure 4) In multivariable analysis, age, GCS of the non-infarcted area, LV mass index, and LV twist were independently correlated with diastolic functional indices. (Table 4) LV mass/volume ratio was not significantly correlated with diastolic functional indices. (r = 0.164, p = 0.326 with E/e’; r = -0.199, p = 0.230 with e’; r = -0.304, p = 0.07 with E/A ratio).

**Table 4:** Univariable and multivariable analyses for diastolic functional indices

	**e’**		**E/e’**		**ED-GLSr**		**ED-GCSr**	
	**Uni-**	**Multi-**	**Uni-**	**Multi-**	**Uni-**	**Multi-**	**Uni-**	**Multi-**
	**R (p-value)**	**β (p-value)**	**R**	**β**	**R**	**β**	**R**	**β**
**Age**	-0.64 (<0.001)	-0.15 (<0.001)	0.52 (0.001)	0.18(0.02)	-0.37 (0.02)	-0.01 (0.048)	-0.42 (0.01)	-0.01 (0.03)
**Male**	0.22(0.18)		-0.21 (0.20)		0.17 (0.32)		0.19 (0.25)	
**Diabetes**	0.32 (0.047)		-0.11 (0.51)		0.22 (0.18)		0.06 (0.73)	
**Hypertension**	-0.36 (0.03)		-0.36 (0.03)	3.05(0.03)	-0.37 (0.02)		-0.04 (0.80)	
**Extent of scar**	-0.27 (0.11)		0.16 (0.35)		-0.14 (0.40)		-0.23 (0.17)	
**LVEF**	0.27 (0.10)		0.06 (0.70)		0.34 (0.04)		0.36 (0.03)	
**LV mass index**	-0.38 (0.02)	-0.06 (0.02)	0.26 (0.12)		-0.31 (0.06)		-0.04 (0.82)	
**M/V ratio**	-0.20 (0.23)		0.16 (0.33)		-0.16 (0.35)		-0.04 (0.83)	
**LVEDWS**	-0.47 (0.003)		0.83(<0.001)†		-0.32 (0.049)		-0.33 (0.046)	
**Twist**	0.39 (0.02)	-0.10 (0.03)	-0.35 (0.03)		0.43 (0.01)		0.70(<0.001) *	
**Non-infarcted myocardial CS**	-0.52 (0.001)	-0.15 (<0.001)	0.30 (0.07)		-0.47 (0.004)	-0.013 (0.01)	-0.73(<0.001)	-0.04(<0.01)

EF = ejection fraction; M/V = LV mass to LV end-diastolic volume ratio; CS = circumferential strain; † Excluded in multivariable analysis for E/e’ and * for ED-GCSr due to significant co-linearity, See abbreviations in the Tables 2 and 3.

### Reproducibility of strains and twist

Intraclass correlation coefficient of GCS, GLS and twist were 0.983 (p < 0.001), 0.880 (p = 0.003), and 0.570 (p = 0.081), respectively. Systolic GCSr and GLSr were 0.728 (p = 0.025) and 0.858 (p = 0.005), respectively. Early diastolic GCSr and GLSr were 0.593 (p = 0.051) and 0.916 (p = 0.001), respectively.

## Discussion

In this study we found that the extent of myocardial scarring and edema were both significantly related to LV systolic function as measured by LV ejection fraction, RWMSI, GLS, and average GCS. In contrast to systolic function, the extent of myocardial injury was weakly or not significantly correlated with LV myocardial and chamber diastolic functional indices. Age, LV mass index, LVEDWS, LV twist, and non-infarcted myocardial circumferential strain were significantly correlated with LV diastolic functional indices. This suggests a significant contribution of age and non-infarcted myocardial characteristics to chamber diastolic function in patients with early reperfused AMI.

### Extent of myocardial injury and systolic function

Our data indicate that the extent of myocardial injury is significantly correlated to all three directional systolic deformational indices (radial wall thickening, longitudinal and circumferential shortening) and LV chamber systolic function. This firmly supports previous data that demonstrated a close relationship between the extent of myocardial injury and myocardial systolic function [16]. However, the extent of myocardial injury was better correlated with GLS rather than GCS as was the case here. This may have resulted from an increased vulnerability to ischemia in the subendocardial fibers which mainly contribute to longitudinal movement; or all of the enrolled subjects may have been successfully reperfused early after AMI and, therefore, the nature of the myocardial infarction tended to be subendocardial. However, the weak relationship observed between LV twist and the extent of myocardial injury suggests that some compensatory hypercontractility in the remaining non-infarcted myocardium or pre-existing myocardial function may also contribute to LV twist after AMI.

### Determinants of diastolic function in AMI

Contrary to the well-established close relationship between the extent of myocardial infarct and systolic function, the relationship between the extent of myocardial injury and chamber diastolic function is controversial [17,18]. Extracellular myocardial components, LV mass, and geometry are known determinants of ventricular compliance [19]. Myocardial edema and infarct have been shown to impair myocardial relaxation and increase myocardial stiffness [20]. However, it requires further study to examine how an area of fibrotic scar tissue affects other segments. Our results indicate that LVEDWS measured by CMR was related to chamber diastolic functional parameters and support the importance of relative wall thickness or mass to LV volume to determine chamber diastolic function. However, as can be seen in this study, the degree of correlation between the extent of myocardial injury and chamber diastolic functional indices was weak. This finding suggests not only the extent of the infarct but also the non-infarcted myocardial characteristics or function significantly affect chamber diastolic function. During and after AMI, compensatory hypercontraction and hyperperfusion has been shown to take place in the remote uninvolved myocardium [21] to maintain stroke volume. However, their effects on chamber diastolic function has not been fully evaluated. According to our study results, the degree of compensatory hypercontractility of remote myocardium may contribute to maintaining diastolic function. This hypothesis is supported in that some young patients could maintain normal LV diastolic function even after AMI. One interesting finding here is that LV twist is significantly related to E/e’, which is representative of LV filling pressure. As LV twist is largely determined by epicardial or midwall mechanics, non-infarcted myocardial compensatory function might significantly contribute to chamber diastolic function in patients with reperfused AMI. We also observed that age was the strongest correlate for diastolic function and was independent of LV ejection fraction and the extent of infarct, which suggests pre-existing myocardial characteristics or contractile reserve before AMI would be important to determining LV chamber diastolic function after AMI, in addition to extent of injury.

### LV twist and chamber diastolic function

Several previous studies have shown that LV twist increases with aging, hypertension, and diabetes in order to compensate for impaired longitudinal function [22-24]. Our study shows that LV twist was significantly correlated to diastolic functional indices, but this correlation was significantly attenuated after adjusting for age and non-infarcted myocardial function. Therefore, a compensatory increase in twist to adapt to decreased regional systolic function may contribute to LV chamber diastolic function. This finding supports the importance of non-infarcted myocardial compensatory function. Aging has previously been associated with concentric remodeling [25,26], subendocardial myofiber dysfunction [27], and reduced elasticity [22], with consequent impaired LV recoil and untorsion [28]. In our study, the greater twist seen in older participants could reflect a compensatory mechanism for decreased myocardial shortening which may help maintain LV ejection fraction and stroke volume. In the modern era of early reperfusion and the resulting lesser extent of myocardial injury, the contributing role of LV twist to diastolic function might be higher.

### Limitations

First, we did not directly measure the tissue characteristics of the non-infarcted myocardium. Instead, we measured myocardial deformation and used this as an index of non-infarcted myocardial regional systolic or diastolic function. Novel imaging method such as T1 mapping may provide additional information on this healthy looking myocardial tissue. Secondly, when measuring circumferential strain of the non-infarcted myocardium, non-LGE or non-edema area could not be exactly matched with echocardiographic segments. We used average values for non-infarcted slices or segments, and the effects of this mismatch are likely small or negligible. Thirdly, when measuring LVEDWS we used tissue Doppler-derived LV end-diastolic pressure instead of an invasive method. This is an unavoidable limitation from using this approach. Fourthly, all the enrolled patients underwent timely, successful PCI resulting in a limited extent of myocardial injury. Therefore, the applicability of this study to all the myocardial infarction patients needs further evaluation. Lastly, using septal e' in patients with regional wall motion abnormalities may limitations but previous studies showed even in regional wall motion abnormality, E/e’ correlated to LV filling pressure, therefore this limitation would not change the results [29].

## Competing interests

The authors declare that they have no competing interests.

## Authors’ contributions

HC and EYC made the study design and wrote the manuscript. CHP, EYC and THK analyzed CMR images, EJK analyzed the speckle tracking echocardiography. HC and JHY collected the echocardiographic and clinical data. YWY, JYK, BKL, PKM, BKH, SJR and,HMK collected the clinical data and angiographic data. All authors read and approved the final manuscript.
